# Novel Polymer Material for Efficiently Removing Methylene Blue, Cu(II) and Emulsified Oil Droplets from Water Simultaneously

**DOI:** 10.3390/polym10121393

**Published:** 2018-12-15

**Authors:** Jie Cao, Jianbei Zhang, Yuejun Zhu, Shanshan Wang, Xiujun Wang, Kaihe Lv

**Affiliations:** 1 School of Petroleum Engineering, China University of Petroleum (East China), Qingdao 266580, China; zhangbeiice@gmail.com; 2 State Key Lab of Offshore Oil Exploitation, Beijing 100028, China; zhuyj3@cnooc.com.cn (Y.Z.); wangshsh24@cnooc.com.cn (S.W.); wangxj89@cnooc.com.cn (X.W.); 3 CNOOC Research Institute Co. Ltd., Beijing 100028, China

**Keywords:** wastewater treatment, adsorption, emulsion separation, thermal crosslinking, superhydrophilicity

## Abstract

The pollution of water resources has become a worldwide concern. The primary pollutants including insoluble oil, toxic dyes, and heavy metal ions. Herein, we report a polymer adsorbent, named SPCT, to remove the above three contaminants from water simultaneously. The preparation process of SPCT contains two steps. Firstly, a hydrogel composed of sulfonated phenolic resin (SMP) and polyethyleneimine (PEI) was synthesized using glutaraldehyde (GA) as the crosslinking agent, and the product was named SPG. Then SPCT was prepared by the reaction between SPG and citric acid (CA) at 170 °C. SPCT exhibited an excellent performance for the removal of methylene blue (MB) and Cu(II) from aqueous solution. For a solution with a pollutant concentration of 50 mg L^−1^, a removal efficiency of above 90% could be obtained with a SPCT dosage of 0.2 g L^−1^ for MB, or a SPCT dosage of 0.5 g L^−1^ for Cu(II), respectively. SPCT also presented an interesting wettability. In air, it was both superhydrophilic and superoleophilic, and it was superoleophobic underwater. Therefore, SPCT could successfully separate oil-in-water emulsion with high separation efficiency and resistance to oil fouling. Additionally, SPCT was easily regenerated by using dilute HCl solution as an eluent. The outstanding performance of SPCT and the efficient, cost-effective preparation process highlight its potential for practical applications.

## 1. Introduction

The increasing emission of organic dyes and heavy metal ions released from the mining, textile, printing, and metal smelting industries, as well as industrial oily wastewater production have caused severe environmental and ecological damage [1]. Many treatment technologies for the removal of this contamination from water have been investigated, including chemical coagulation, photo-degradation, filtration, ion exchange, adsorption, and electrochemical treatments [2]. Among these methods, adsorption is generally considered to be the most convenient and effective approach because it is widely applicable and economically favorable [3]. However, few separation methods can simultaneously purify the three types of pollutants. Therefore, it is crucial to develop novel materials to deal with complicated wastewater.

There have been many reports on the removal of multiple pollutants in wastewater treatment systems, but only a few of them mention the adsorption of insoluble oil at the same time. Yang et al. synthesized a perfluorous conjugated microporous polymer which realizes efficient removal all three types of pollutants from water [4]. But this kind of materials are recycled through being washed by organic solvent after adsorbing oil, which is not economical or environmentally friendly. Qian et al. prepared a membrane which consisted of a TiO_2_ nanorod array mesh, integrated with a sulfonated graphene oxide/Ag nanoparticle layer for separating oil/water emulsions and degrading soluble organic contaminants in one step [5]. But UV irradiation is costly, consumes significant energy and leads to a low application possibility. To the best of our knowledge, there are few reports on the adsorbents which can efficiently remove insoluble oil, soluble organic molecules and heavy metal ions from water, simultaneously.

Polyethyleneimine (PEI), as a typical water-soluble polyamine, contains primary, secondary and tertiary amine groups on its molecular chain, which are effective functional groups to improve adsorption capacity [6,7]. These active amine groups can be crosslinked by an aldehyde or epoxy group to fabricate hydrogels, membranes, and fibers for adsorption, gene delivery, etc. [8,9]. Several studies have confirmed that PEI-based materials have an effect on wastewater treatment systems [10]. On the other hand, sulfonated phenolic resin (SMP) plays an important role in deep well drilling. SMP-based drilling fluid systems have become the major system for the oil and gas industry, especially in high temperature and salinity environments [11]. SMP contains abundant phenyl groups and oxygen-containing functional groups, such as –OH and –SO_3_^−^, which have been demonstrated to be efficient adsorption sites. For example, Wei et al. have prepared a sulfonic-functionalized graphene oxide via a temperate and easily handled method, this material showed a high adsorption behavior for heavy metal ions and dyes [12].

For emulsion separation, especially for the separation of surfactant-stabilized emulsions, polymer dominated membranes have been found to be a progressive method with a simple operation process [13]. In order to improve the separation property and stability of polymeric membranes, a lot of approaches, such as surface modification, blending with inorganic nanoparticles and other additives, and polymer grafting have been applied [14,15,16,17]. However, performance improvements are still severely limited, remaining far below applicable requirements. Therefore, using special wettability to design novel materials is an effective and facile way of determining the applicability of materials to wastewater remediation. Jiang et al. proposed a new concept, which took advantage of high-surface-energy materials having water-favoring properties, to construct underwater superoleophobic materials to separate oil droplets from water [18]. As water usually has higher density than oil, for a solid with a superhydrophilic surface in air and a superoleophobic surface in water, a water-barrier layer will form on its surface, thus effectively preventing oil pollution of the solid during oil/water separation. To enhance the hydrophilicity of polymer materials, the introduction of hydrophilic groups on the material surface is necessary. Thus, a lot of hydrophilic compounds, such as poly(acrylic acid), acrylic acid, polyacrylamide and poly(dimethylamino ethyl methacrylate) have been grafted on polymer matrices [19,20,21].

Herein, we report a novel polymer adsorbent, named as SPCT, to remove toxic dyes, heavy metal ions and emulsified oil droplets from water, simultaneously. Firstly, a crosslinked hydrogel, named as SPG was synthesized by the reaction between SMP, PEI and glutaraldehyde (GA), and SPCT was prepared by the reaction between SPG and citric acid (CA) under high temperature. SPCT presented an excellent performance for the removal of methylene blue (MB) and Cu(II) from aqueous solution. Moreover, SPCT exhibited superhydrophilicity and superoleophilicity in air and superoleophobicity under water. This special wettability allowed SPCT to be reutilized to separate a Span 80-stabilized toluene-in-water emulsion with high efficiency. As such, our research demonstrates the development of a simple, inexpensive, and efficient material, which shows great application potential for wastewater treatment.

## 2. Materials and Methods

### 2.1. Materials

Sulfonated phenolic resin (SMP) was obtained from Dafang Synthetic Chemical Co. Ltd. (Chongqing, China). Polyethyleneimine (PEI) with a molecular weight of 1800 and glutaraldehyde (GA) with a concentration of 50 wt % were purchased from Shanghai Aladdin Biochemical Technology Co. Ltd. (Shanghai, China). Other chemical reagents of analytical grade, including citric acid (CA), methylene blue (MB), CuSO_4_·5H_2_O, Pb(NO_3_)_2_, polyoxyethylene sorbitan fatty acid esters (Span 80) and toluene were all purchased from Shanghai Sinopharm Chemical Reagent Co. Ltd. (Shanghai, China). Crystal violet (CV), congo red (CR), methyl orange (MO) were purchased from Tianjin Oubokai Chemical Reagent Co. Ltd. (Tianjin, China). Deionized water was used throughout the experiments. All reagents were obtained from commercial sources and used without further purification.

### 2.2. Preparation of SPCT

A mixture of SMP (1.0 g), PEI (1.0 g) and NaOH (0.3 g) was dissolved in deionized water (20 mL) and stirred continuously to form a homogeneous solution. Then GA (1 mL) was slowly added into the solution. The mixture was then kept at 60 °C for 1 h under constant stirring. After being cooled down to room temperature, the dispersed mixture was sonicated and the product was named SPG. After that, 10 mL of CA solution (15 wt %) was added to the SPG dispersion under stirring and the system was kept in a hot water bath at 60 °C for 2 h. The system was dried in vacuum oven at 60 °C, then transferred into an electric thermostatic oven at 170 °C for 2 h to conduct the thermal crosslinking. The product was sieved between 50 mesh and 40 mesh to obtain the adsorbent (SPCT).

### 2.3. Characterization

The Fourier transform infrared (FTIR) spectrum was recorded on a Tensor 27 spectrometer (Bruker, Zurich, Switzerland) from 4000–400 cm^−1^ at a resolution of 4 cm^−1^ with a total of 16 scans using powder-pressed KBr pellets at room temperature. The thermal characteristics of the samples were obtained using a 209F3 thermogravimetic analysis (TGA) system (NETZSCH, Bavaria, Germany) in the range of 30–800 °C at a heating rate of 10 °C min^−1^ under a nitrogen atmosphere. Optical microscopy images of emulsions were taken on an XS-2100 microscope (Yongxin, China) by dropping emulsion solution on a glass slide. Contact angles were measured by an OCA-20 system (Data-physics, San Jose, CA, USA) equipped with video capture at room temperature. The droplet volume was 2 µL, and the average value was obtained from three measurements per sample. Toluene was used as the oil phase, and distilled water, NaCl solution (10 wt %), HCl solution (1 mol L^−1^) or NaOH solution (1 mol L^−1^) was used as the water phase for different experiments. The equilibration time for the movement of droplets on the adsorbent surface was 5 min.

### 2.4. Batch Adsorption Experiments

All batch adsorption experiments were performed on a model DF-101Z magnetic stirrer (Yuhua, Zhengzhou, China) with a stirring speed of 120 rpm. To study the adsorption kinetics, 0.01 g of SPCT was added into 50 mL of aqueous dye solution with the initial concentration of 50 mg L^−1^ for different time intervals at 25 °C. For the adsorption of heavy metal ions, the amount of adsorbent was 0.025 g. The solution pH was adjusted by adding a negligible amount of 1 mol L^−1^ HCl and 1 mol L^−1^ NaOH to examine the effect of different pH values on the adsorption capacity. Adsorption isotherms were conducted with initial contaminant concentrations ranging from 0 to 200 mg L^−1^. The concentration of dye in the supernatant solution was determined by using a UV-1780 spectrophotometer (Shimadzu, Kyoto, Japan) based on the standard curve. The concentration of heavy metal ion in the supernatant solution was determined by an atomic absorption spectrophotometer (Varian Spectra HP 3510, Santa Clara, CA, USA). The adsorption capacity was expressed as the following equation:

(1) $$q=\frac{\left(C_{i}-C_{t}\right)V}{m}$$

where *q* is the amount of adsorption capacity at time *t* (mg g^−1^), *C_i_* is the initial concentration of contaminant (mg L^−1^), *C*_t_ is the contaminant concentration at time *t* (mg L^−1^), *V* is the volume of the solution (L), and *m* is the mass of the adsorbent (g).

### 2.5. Separation of Oil-In-Water Emulsion

For the oil-in-water emulsion, 99 g of water, 1 g of toluene and 0.02 g of Span 80 were mixed and mechanically stirred for 30 min to form the oil-in-water emulsion. The average size of dispersed toluene droplets was about 500 nm. The device for the emulsion separation is shown in Figure 9a. The SPCT powder (1.0 g) was evenly adhered between two sheets of filter paper having a radius of 2.5 cm and fixed between two glass tubes. Then, 30 mL of the oil-in-water emulsion was poured into the glass tube. The entire separation process was solely driven by a vacuum pump operating at a pressure of 0.1 MPa. The toluene content in water was measured by using the UV-1780 spectrophotometer at 261 nm. The separation efficiency was defined and calculated by the oil rejection coefficient *R* (%) according to the equation:

(2) $$R\left(\%\right)=\left(1-\frac{C_{P}}{C_{0}}\right)\times 100\%$$

where *C*_0_ and *C_P_* (mg L^−1^) are the oil concentration in the original toluene-in-water mixture and the collected water, respectively.

### 2.6. Regeneration and Reusability Experiments

The regeneration of SPCT was evaluated by checking the cycle number dependence of the adsorption capacity for MB or Cu(II) solution (50 mg L^−1^). The adsorbent loaded with MB or Cu(II) was added to HCl solution (1 mol L^−1^), and the mixture was shaken for 15 min. Then, the regenerated adsorbent was washed with water three times for the next adsorption. The desorption efficiency was calculated using the following expression:

(3) $$Desorption\;efficiency\;\left(\%\right)=\frac{C_{i}}{C_{0}}\times 100\%$$

where *C*_0_ and *C*_i_ (mg L^−1^) are the original and the residual concentrations of MB or Cu(II), respectively.

In order to test the reusability for the separation of toluene-in-water emulsion, SPCT was reused directly after being dried at 60 °C for the next emulsion separation.

### 2.7. Simultaneous Removal of Three Pollutants

Three pollutants including a toluene-in-water emulsion (1 wt %), MB (50 mg L^−1^) and Cu(II) (50 mg L^−1^) were in one solution. The separation process is the same as described in Section 2.5. The volume of pollutant solution ranged from 40 to 200 mL.

## 3. Results

### 3.1. Characterization of SPCT

**Figure 1:**
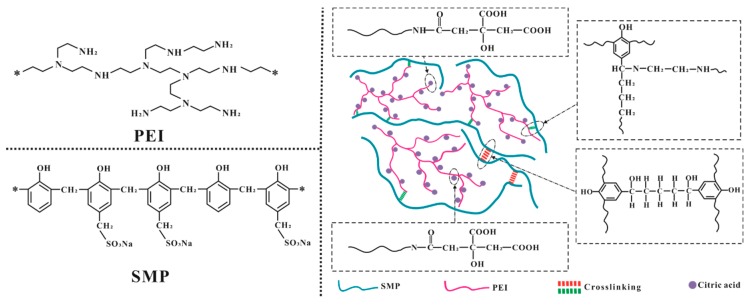
Molecular structure of polyethyleneimine (PEI), sulfonated phenolic resin (SMP) and the adsorbent (SPCT).

**Figure 2:**
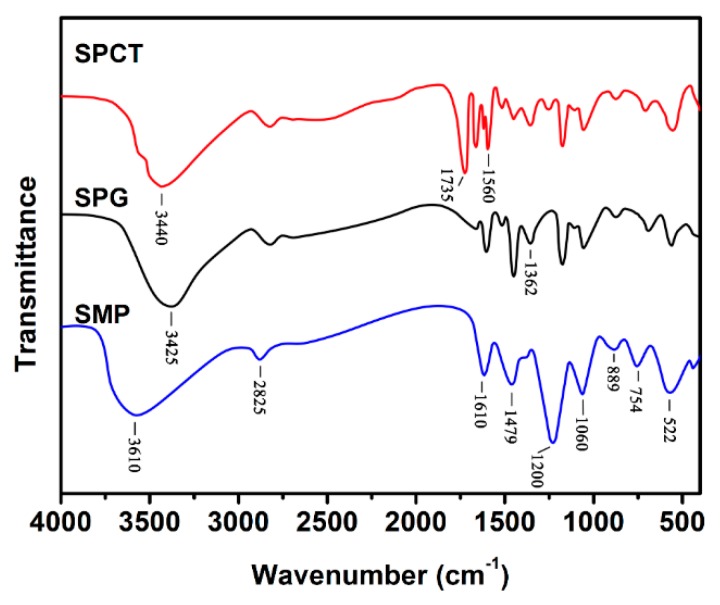
FTIR spectrum of SMP, SPG and SPCT.

The molecular structures of SMP, SPG and SPCT are illustrated in Figure 1, and they were confirmed by FTIR analysis as shown in Figure 2. For SMP, the peaks at 522, 1060 and 1200 cm^−1^ could be attributed to the absorption band of sulfonic groups, the peaks at 754, 889 and 1610 cm^−1^ were assigned to the absorption band of phenyl groups, and the peaks at 1479 and 2825 cm^−1^ were due to the bending vibration and stretching vibration of C–H. The broad peak above 3200 cm^−1^ is classically attributed to the stretching vibration of O–H or N–H bond, and the O–H bond usually absorbs at a high wavenumber. As a result, the movement toward the low wavenumber for this broad peak is observed for SPG and SPCT due to the presence of the PEI component. Moreover, the introduction of the PEI component in SPG and SPCT can also be proven by the peak at 1362 cm^−1^, which is attributed to the stretching vibration of C–N bond [22]. Additionally, two new peaks at 1600 and 1735 cm^−1^ can be attributed to the absorption of the carbonyl groups. The peak at 1600 cm^−1^ is the evidence for the presence of amide groups, which indicates the thermal crosslinking between CA and PEI. Compared to the carbonyl groups in amide or ester structures, the carbonyl groups of carboxylic acid in an acidic form absorb at a high wavenumber, as a result, the peak at 1735 cm^−1^ indicates the presence of carboxylic acid structure in SPCT [23].

**Figure 3:**
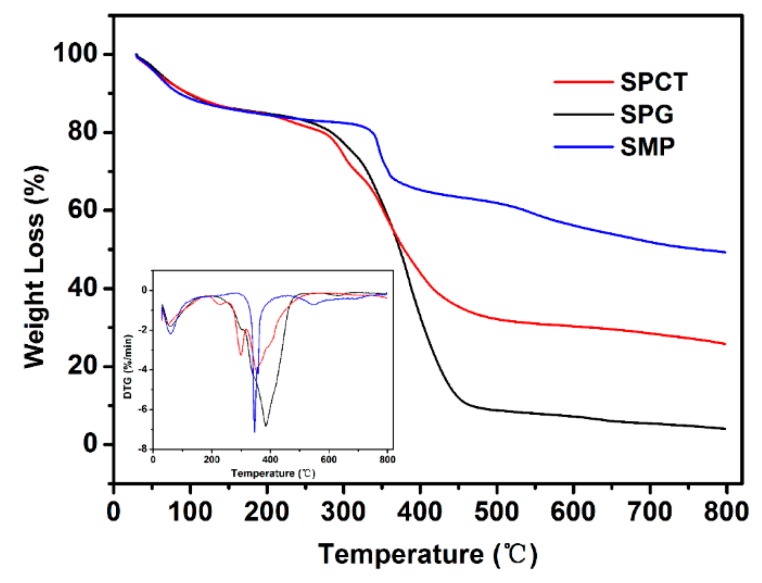
Thermogravimetic analysis (TGA) curve of SMP, SPG and SPCT.

The thermal characteristics of SMP, SPG and SPCT were examined with TGA, and the results are shown in Figure 3. There is a mass-loss peak before 100 °C for each sample, and this peak is usually attributed to the evaporation of small molecules in the sample. SMP presents the best thermal stability and it had mainly two mass-loss peaks at around 346 and 547 °C. Among the three samples, SMP also had the greatest residual weight percentage at 800 °C due to it having the highest content of negatively charged groups. Unlike this, four main mass-loss peaks at around 302, 340, 383 and 637 °C were observed for SPG, and the primary mass-loss peak at 383 °C could be attributed to the decomposition of PEI component because of its high content in SPG [24]. Compared to the above two samples, the thermal decomposition process of SPCT was much more complicated. This might have been caused by the introduced carboxylic groups, amide groups and ammonium carboxylate structures after the thermal crosslinking between SPG and CA.

### 3.2. Adsorption of Dyes and Heavy Metal Ions

#### 3.2.1. Effect of Pollutant Type on the Adsorption Capacity of SPCT

**Figure 4:**
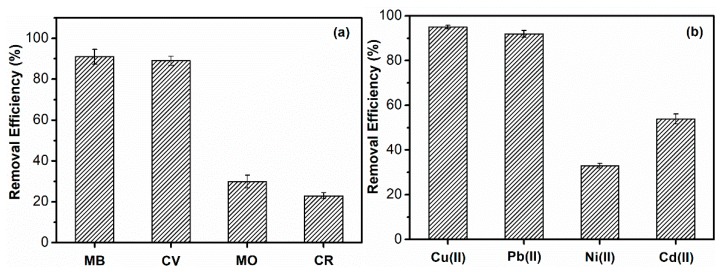
The adsorption efficiency of removing (**a**) dyes and (**b**) heavy metal ions by SPCT.

In order to screen out the best adsorbate for SPCT, two anionic dyes, such as CR and MO, cationic dyes, such MB and CV, and four metal ions, such as Cu(II), Pb(II), Ni(II) and Cd(II), were investigated, and the results are shown in Figure 4. For the two cationic dyes, SPCT presents remarkable adsorption performance and each removal efficiency is about 90%. However, the removal efficiencies for MO and CR are 30.3% and 23.2%, respectively. In general, the interaction between adsorbent and dye molecules contains π-π interactions, hydrogen bond, hydrophobic and electrostatic interactions [25,26,27]. The structure difference in these dyes could affect the strength of the interaction between adsorbent and adsorbate, as a result, a significant difference in removal efficiencies could be observed. For the removal of metal ions, the removal efficiency happens in the order of Cu(II) (95.4%) > Pb(II) (91.8%) > Cd(II) (54.2%) > Ni(II) (33.6%). Similar results have been reported by other researchers. For example, PEI-based adsorbent exhibited great adsorption capacity for Cu(II) and Pb(II) not only due to the amine groups which were involved in the chelation interaction during the adsorption process, but also the larger binding constant of PEI to Cu(II) ions than that of other metal ions [28]. In addition, SMP-based adsorbents exhibited a good ion-exchange mechanism on Pb(II), and sulfonic and phenolic groups were probably involved in the sorption process as well [29]. As a result, MB and Cu(II) are selected as pollutant examples for the following research.

#### 3.2.2. Effect of Dosage on the Removal Efficiency of SPCT

**Figure 5:**
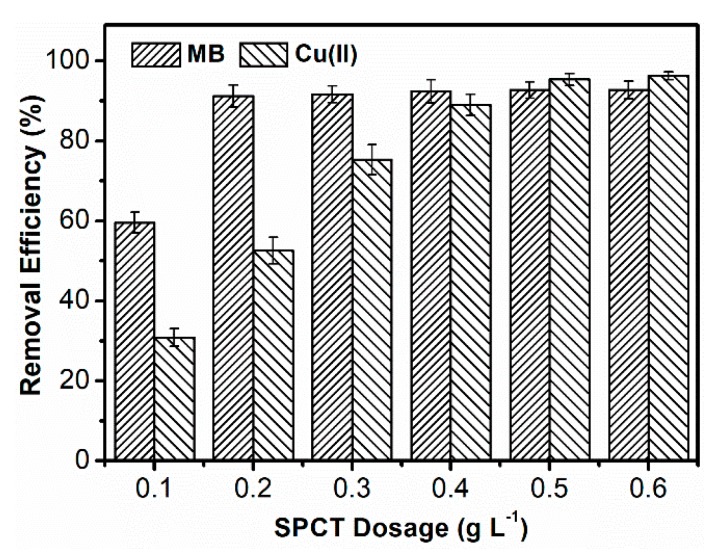
Effect of SPCT dosage on the adsorption of MB and Cu(II).

In a wastewater treatment process, the proper dosage of an adsorbent is one of the important factors to improve the removal efficiency and reduce the cost. The effect of SPCT dosage on the removal efficiency for MB and Cu(II) was studied as shown in Figure 5. With the increase of SPCT dosage, the removal efficiency increased sharply for both pollutants at low dosage, and the removal efficiency reached a balanced value when the dosage was above a critical value. It is found that, if the removal efficiency exceeded 90% as a standard, the critical SPCT dosages were 0.2 g L^−1^ and 0.5 g L^−1^ for the removal of MB and Cu(II), respectively. Compared to the dosages of other materials for wastewater treatment, SPCT was an efficient adsorbent for the two pollutants [30,31]. Furthermore, the dosage of 0.2 g L^−1^ for the dye treatment and the dosage of 0.5 g L^−1^ for the metal ion treatment were used in the following research.

#### 3.2.3. Adsorption Kinetics

**Figure 6:**
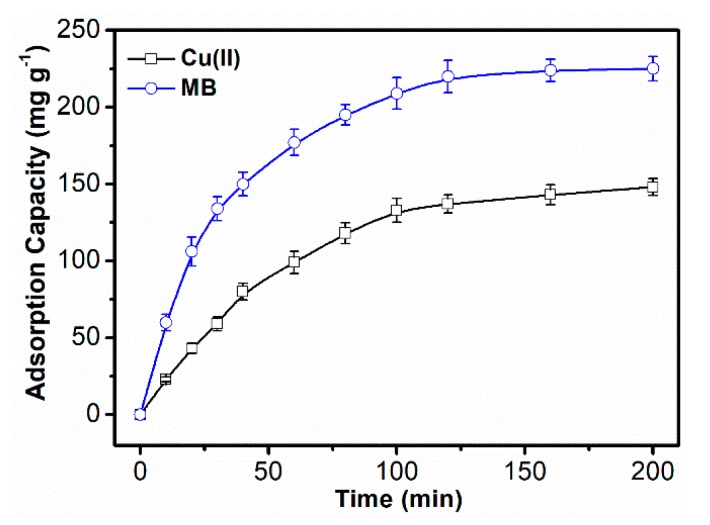
Adsorption kinetics curve of the adsorption of MB and Cu(II).

Figure 6 shows that the adsorption kinetics processes of MB and Cu(II) were similar. The adsorption capacity increased rapidly in the initial 50 min and reached equilibrium in approximately 120 min. The reason for the obvious reduction in adsorption rate as the adsorption time increased is that the available adsorption sites gradually decreased. The equilibrium adsorption capacities for MB and Cu(II) obtained after 150-min adsorption were 225 mg g^−1^ and 148 mg g^−1^, respectively.

To further investigate the adsorption kinetics, the obtained data were formulated by the pseudo-first-order model (Equation (4)) and pseudo-second-order model (Equation (5)):

(4) $$log\left(q_{e}-q_{t}\right)=logq_{e}-\frac{K_{1}t}{2.303}$$

(5) $$\frac{t}{q_{t}}=\frac{t}{q_{e}}+\frac{1}{K_{2}q_{e}^{2}}$$

where *q_e_* and *q_t_* (mg g^−1^) is the adsorption capacity in equilibrium time and at any time *t* (mg g^−1^), and *K*_1_ (min^−1^) and *K*_2_ (g mg^−1^ min^−1^) are the pseudo-first-order and pseudo-second-order rate constants, respectively [10].

The corresponding kinetic adsorption parameters calculated from the above two kinetic models are listed in Table 1. The determination coefficient *R*^2^ (0.995 and 0.997) of the pseudo-first-order model is much greater than that *R*^2^ (0.993 and 0.978) of the pseudo-second-order model, so the adsorption process of MB and Cu(II) can be described well with the pseudo-first-order model.

#### 3.2.4. Adsorption Isotherm

**Figure 7:**
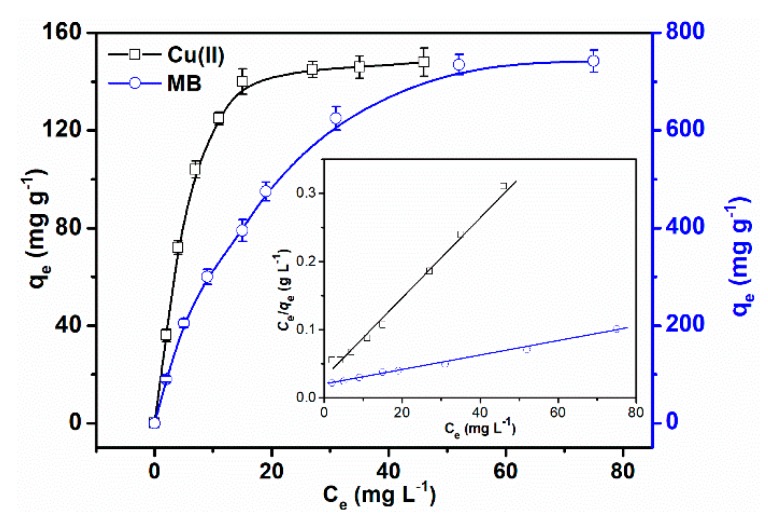
Adsorption isotherm curve of the adsorption of MB and Cu(II) (inset, Langmuir isotherm plots).

As shown in Figure 7, the adsorption isotherm was carried out to get a better understanding of the adsorption property of SPCT. There are two main models that describe adsorption behavior, namely the Langmuir and the Freundlich isotherm models.

The Langmuir model is defined as:

(6) $$\frac{C_{e}}{q_{e}}=\frac{C_{e}}{q_{m}}+\frac{1}{K_{L}q_{m}}$$

The Freundlich model is defined as:

(7) $$q_{e}=K_{F}C_{e}^{n}$$

where *C_e_* is the equilibrium concentration of the adsorbate in solution (mg L^−1^); *q_e_* is the equilibrium adsorption capacity (mg g^−1^); *q_m_* is the maximum adsorption capacity of the adsorbent (mg g^−1^); *n* is an empirical parameter in the Freundlich model; and *K_L_* and *K_F_* are the binding constants of the Langmuir equation and the Freundlich equation, respectively [32].

The regression adsorption isotherm parameters of MB and Cu(II) by the Langmuir and the Freundlich models are listed in Table 2. Determined by the correlation coefficient *R*^2^, the adsorption equilibrium isotherm of MB and Cu(II) are described better with the Langmuir model than the Freundlich model, suggesting that the adsorption of MB and Cu(II) is a homogeneous and monolayer adsorption process [22]. And *K_L_* value is larger than 0 and smaller than 1 which suggests that adsorption is favorable [29]. The calculated maximum adsorption capacities of MB and Cu(II) are 742 mg g^−1^ and 148 mg g^−1^ according to the Langmuir model.

#### 3.2.5. Effect of Solution pH on the Adsorption Capacity of SPCT

**Figure 8:**
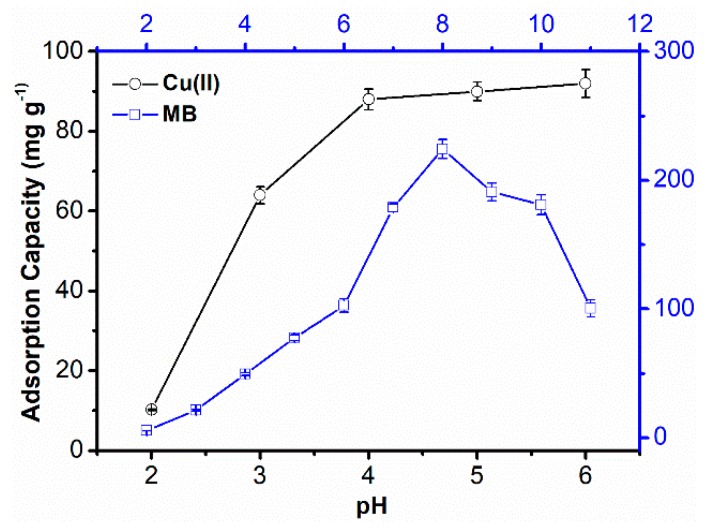
Effect of pH value on the adsorption capacity of MB and Cu(II).

The pH value of a solution has a great influence on both the ionization of dye molecules and the structure of adsorbent, as well as the form of metallic species in solution, which would lead to huge adsorption differences. As shown in Figure 8, it can be observed that the adsorption capacity of MB was highest at a pH of 8 and decreased with further increases or reductions in pH. This phenomenon can be explained by the electrostatic action between MB molecules and SPCT components. At acidic pH, the amine groups of SPCT and the dimethylamine groups from MB are almost protonated as positively charged ammonium structures, therefore the electrostatic repulsion between MB and SPCT restricts the adsorption. In a highly basic environment, the low adsorption capacity of MB could be due to the increase in the density of electron clouds for groups containing nitrogen atom, and this also promotes the repulsion interaction between MB and SPCT [32]. The adsorption capacity towards Cu(II) was quite different with MB. At a low pH, the protonation of the amine groups is dominant, and thus enhances electrostatic repulsion to the same positively charged Cu(II) ions, resulting in a very low adsorption capacity. When the pH is above 4, the adsorption capacity of Cu(II) is slightly influenced by the pH value. For these two pollutants, the insignificant adsorption capacities were found at a low pH value, and this means using a dilution inorganic acid solution as the eluent could be an efficient method for the regeneration of SPCT both in the non-competitive and competitive removal of MB and Cu(II).

### 3.3. Separation of Oil-In-Water Emulsion

**Figure 9:**
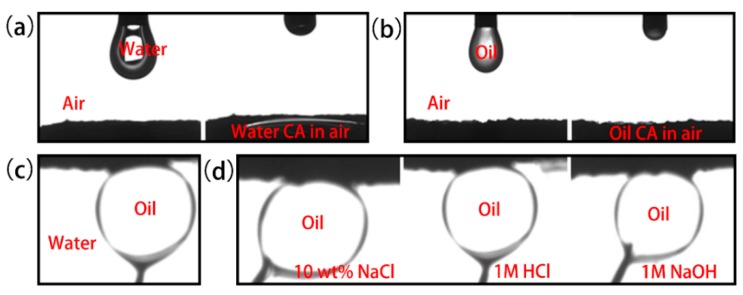
The wetting process of (**a**) a water droplet and (**b**) a toluene droplet on the surface of SPCT in air. (**c**) The toluene contact angle of SPCT in water. (**d**) The contact angle pictures of toluene in aqueous NaCl (10 wt %), HCl (1 mol L^−1^) and NaOH (1 mol L^−1^) solutions.

The wettability of solid has a decisive influence on the separation of oil-in-water emulsion, as a result, the wettability of SPCT was examined in air and in water [33]. Figure 9a,b show the wetting behavior of water and toluene on the surface, in air. When a water or toluene droplet (2 μL) comes into contact with the surface of SPCT, it immediately spreads out and permeates into the surface, and a contact angle (CA) of nearly 0° is obtained. Both processes are completed within 1 s, fully suggesting the high affinity to water and toluene of the surface in air. Moreover, the surface of SPCT became superoleophobic once immersed in water (Figure 9c). The underwater toluene droplet attained quasi-spherical shape on the surface with a contact angle of 157°. Actually, the environment of emulsion separation is always hard, the oleophobic stability of the SPCT surface in corrosive liquids was also tested. As shown in Figure 9d, the toluene droplet showed a quasi-spherical shape on the surface with an toluene contact angle of 153°, 151°, 155° in NaCl (10 wt %) solution, HCl (1 mol L^−1^) solution and NaOH (1 mol L^−1^) solution, respectively, which indicates the stable superoleophobicity of SPCT in a variety of corrosive liquids.

In the three-phase (oil, water and solid) system, the special wettability is due to the high surface energy and large number of hydrophilic groups on solid surface. In water, SPCT was completely infiltrated by water due to its super hydrophilicity. When it comes into contact with an oil droplet, a three-phase interface can be created because high level of water is trapped in its rough microstructure. As a result, the trapped water will greatly reduce the contact area between oil and solid surface, resulting in a large oil contact angle in water (superoleophobicity) [34].

**Figure 10:**
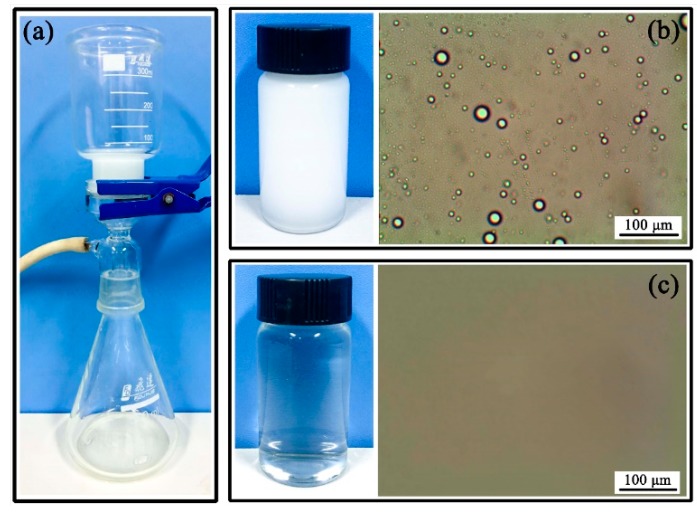
(**a**) The digital image of the setup for separating toluene-in-water emulsion. The photographs and microscopic images of toluene-in-water emulsion (**b**) before and (**c**) after filtration.

Figure 10 gives the separating result of the Span80-stabilized toluene-in-water emulsion. The original emulsified oil is shown in Figure 10b, where dense toluene droplets were clearly observed. However, after passing through SPCT, these droplets were no longer observed in filtrate solution, implying that the toluene in emulsion had been successfully removed. The content of the toluene was calculated by measuring the toluene weight percentage in the filtrate solution, and the removal efficiency of toluene was 98.9%, which indicates a great separation capacity.

### 3.4. Reusability of SPCT

**Figure 11:**
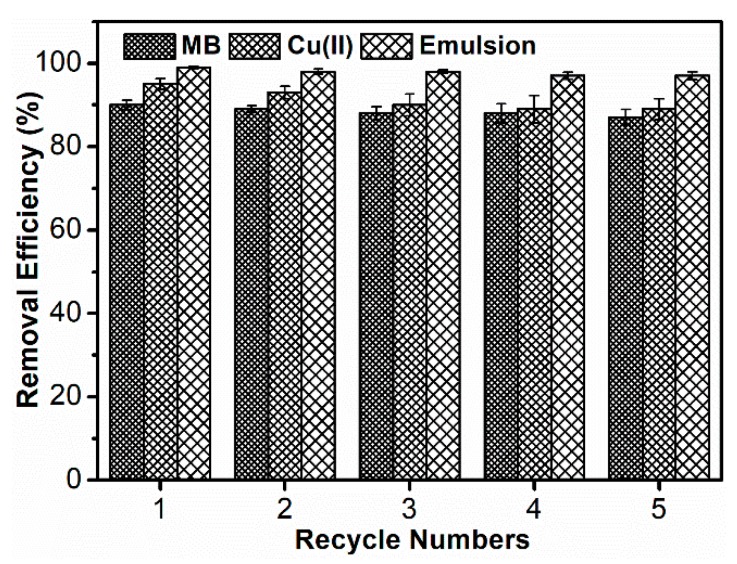
Removal efficiency for different adsorption-desorption cycles.

The regeneration and recyclability experiments were conducted over five cycles, and the results are shown in Figure 11. Desorption studies can help to elucidate the mechanism for an adsorption process. If the adsorbed dye molecules can be desorbed by water, it can be concluded that the interaction between the dye molecule and the adsorbent is due to a weak bond. In other situations, strong acids, such as HCl, can be used as an eluent for the desorption of dye molecules. This means the attachment of the dye molecules onto the adsorbent is by ion exchange or electrostatic attraction [35]. The adsorption capacity towards MB had big differences with different pH values, which endows its excellent adsorption-desorption capacity. As a result, a dilute HCl solution (1 mol L^−1^) was chosen as the eluent for the regeneration of SPCT after adsorbing MB. After the fifth cycle, the removal efficiency was 87.2%.

Different chemicals such as acetic acid, HCl and EDTA salts were employed as eluents for the regeneration of SPCT. Among the reagents, HCl (1 mol L^−1^) exhibited superior performance for the regeneration after Cu(II) adsorption. At the end of the fifth cycle, SPCT retained 89.0% of its original adsorption capacity. The removal efficiency was slightly lower after each cycle because Cu(II) could not be incompletely desorbed. This could be due to loosing of some functional groups of the SPCT by acid cleavage [36].

SPCT was just dried before the next use during the separation of oil-in-water emulsions. However, after five times of filtration, the removal efficiency of SPCT kept stable with separation efficiency exceeding 98%. The reason why SPCT showed such efficient separation was that when the hydrophilic surface contacts with the emulsion, water could be trapped, which greatly decreased the contact area between oil droplet and solid surface [18]. The impacts of oil, water and surfactant on the molecular structure of SPCT was insignificant. As a result, SPCT could separate emulsions with high efficiency and high durability.

### 3.5. Simultaneous Removal of Three Pollutants

**Figure 12:**
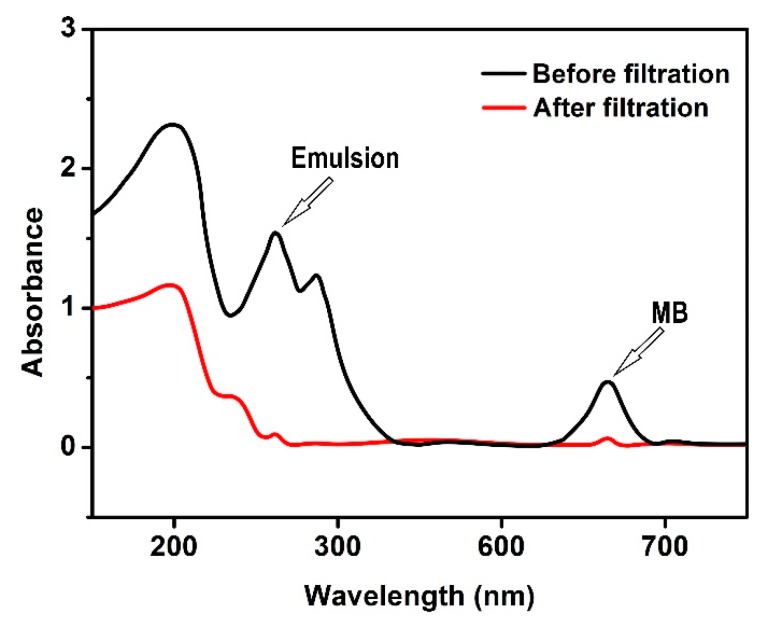
The UV-Vis spectrum of pollutant solution before and after filtration.

**Figure 13:**
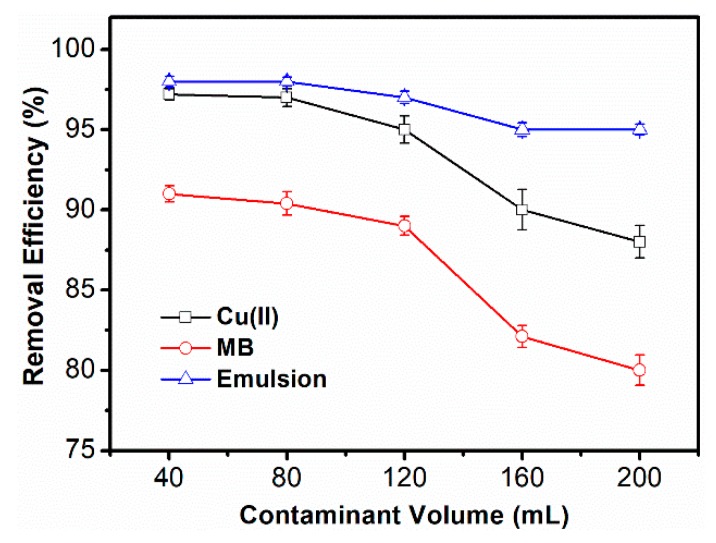
Effect of solution volume on the removal ability of MB, Cu(II) and emulsion.

As shown in Figure 12, it is demonstrated that SPCT could simultaneously remove the three pollutants mentioned above. After the treatment for 40 mL of pollutant solution, the UV-Vis spectrum of filtrated water shows that the band of MB (664 nm) decreased to nearly zero and the band of toluene (261 nm) substantially disappeared. The removal efficiencies of toluene, MB and Cu(II) were 98%,91% and 97%, respectively. The continuous separation ability of SPCT for mixed contaminant solution was also tested as shown in Figure 13. When the solution volume was less than 80 mL, the removal efficiencies for all three pollutants were basically unchanged with the increase of solution volume. However, a noticeable reduction in the removal efficiency could be observed for each pollutant when the solution volume was more than 120 mL. Among the three pollutants, the removal of toluene was found to be the most efficient. This is consistent with the simplest method of the regeneration after emulsion separation. At the volume of 200 mL, the three-phase removal rate could still be maintained above 80%, which means SPCT is an efficient material for the simultaneous removal of pollutants from aqueous solution.

## 4. Conclusions

In summary, a novel polymer material with various functional groups, such as phenyl, hydroxyl, amine, amide, carboxylate, sulfonate groups, was synthesized using the thermal modification of SPG by CA. SPCT exhibited an excellent performance for the removal of certain cationic dyes, such as MB and CV, and heavy metal ions, such as Cu(II) and Pb(II), from aqueous solution at a relatively low dosage. SPCT showed stable superoleophobicity under water which endowed it with a good performance in separating oil-in-water emulsions. The results for the simultaneous removal of MB, Cu(II) and toluene droplets from aqueous solution indicated that SPCT could efficiently separate the three pollutants, which implies its potential application for actual wastewater. SPCT is not only low-cost to be synthesized, easy to be regenerated and efficient for adsorption, but also provides a sustainable approach for oil-in-water emulsion separation. It may lead to progress in economical water purification.

## Figures and Tables

**Table 1:** Kinetics parameters for the adsorption of MB and Cu(II).

Adsorbate	Pseudo-First-Order Kinetic Model			Pseudo-Second-Order Kinetic Model		
	*K*_1_ (min^−1^)	*q_e_* (mg g^−1^)	*R* ^2^	*K*_2_ (g mg^−1^ min^−1^)	*q_e_* (mg g^−1^)	*R* ^2^
MB	0.0292	223	0.995	1.27 × 10^−4^	264	0.993
Cu(II)	0.0292	155	0.997	6.82 × 10^−5^	209	0.978

**Table 2:** Parameters of the Langmuir model and the Freundlich model for the adsorption of MB and Cu(II).

Adsorbate	Langmuir			Freundlich		
	*K_L_* (L mg^−1^)	*q_m_* (mg g^−1^)	*R* ^2^	*K_F_* (mg^1−n^ L^n^ g^−1^)	*n*	*R* ^2^
MB	0.053	952	0.992	74.41	0.587	0.952
Cu(II)	0.209	167	0.991	37.71	0.446	0.798
